# Dynamic Neuro-Cognitive Imagery (DNI^TM^) Improves Developpé Performance, Kinematics, and Mental Imagery Ability in University-Level Dance Students

**DOI:** 10.3389/fpsyg.2019.00382

**Published:** 2019-03-01

**Authors:** Amit Abraham, Rebecca Gose, Ron Schindler, Bethany H. Nelson, Madeleine E. Hackney

**Affiliations:** ^1^Department of Medicine, Division of General Medicine and Geriatrics, Emory University School of Medicine, Atlanta, GA, United States; ^2^Department of Kinesiology, College of Education, University of Georgia, Athens, GA, United States; ^3^Department of Dance, Franklin College of Arts and Sciences, University of Georgia, Athens, GA, United States; ^4^Department of Mathematics, The Weizmann Institute of Science, Rehovot, Israel; ^5^Atlanta VA Center for Visual and Neurocognitive Rehabilitation, Decatur, GA, United States

**Keywords:** mental imagery, dance, range-of-motion, dynamic neuro-cognitive imagery, training, developpé, kinematics

## Abstract

Dance requires optimal range-of-motion and cognitive abilities. Mental imagery is a recommended, yet under-researched, training method for enhancing both of these. This study investigated the effect of Dynamic Neuro-Cognitive Imagery (DNI^TM^) training on developpé performance (measured by gesturing ankle height and self-reported observations) and kinematics (measured by hip and pelvic range-of-motion), as well as on dance imagery abilities. Thirty-four university-level dance students (*M* age = 19.70 ± 1.57) were measured performing three developpé tasks (i.e., 4 repetitions, 8 consecutive seconds hold, and single repetition) at three time-points (2 × pre-, 1 × post-intervention). Data were collected using three-dimensional motion capture, mental imagery questionnaires, and subjective reports. Following the DNI^TM^ intervention, significant increases (*p* < 0.01) were detected in gesturing ankle height, as well as in hip flexion and abduction range-of-motion, without significant changes in pelvic alignment. These gains were accompanied by self-reported decrease (*p* < 0.05) in level of difficulty experienced and significant improvements in kinesthetic (*p* < 0.05) and dance (*p* < 0.01) imagery abilities. This study provides evidence for the motor and non-motor benefits of DNI^TM^ training in university-level dance students.

## Introduction

Dance is an art combining physical, cognitive, and social skills. Technical mastery and physiological elements such as range of motion (ROM) are necessary for dancers to achieve artistic and aesthetic competence (Deighan, 2005; Brown et al., 2007; Angioi et al., 2009). Therefore, dance students constantly aim to increase active, functional ROM for higher performance in meeting choreographic demands (Bronner and Ojofeitimi, 2011; Abraham et al., 2016, 2017). Specifically, increasing hip joint ROM is considered of high importance (Bennell et al., 1999, 2001; Deighan, 2005; Bronner and Ojofeitimi, 2006, 2011; Debarnot et al., 2014). Psychological-cognitive elements such as body-awareness, concentration, and self-confidence have also been suggested to be relevant for enhancing dance performance and ROM (Franklin, 2014, 2019; Fish et al., 2004; Mainwaring and Krasnow, 2010; Abraham et al., 2016, 2017). Specifically, proprioceptive awareness has been suggested as an important factor for dance motor skill (Gamboian et al., 2000; Kiefer et al., 2013).

Being highly motivated to improve their dance performance and ROM, dance students use a variety of strategies (e.g., pelvic hiking or “tucking under”), some of which can lead to injuries (Hagins, 2011). Dance students sustain high incidence of injuries (Garrick, 1999; Askling et al., 2002; Bronner and Ojofeitimi, 2011), with up to 76% of all injuries among dance students aged 10–21 occurring in the lower extremities (Leanderson et al., 2011). Such injuries are thought to be mostly overuse in nature (Bronner and Worthen, 1999; Solomon et al., 1999) and are linked to, among others, alignment, technique, postural faults (Contompasis, 1984; Khan et al., 1995; Garrick, 1999; Luke et al., 2002; Gamboa et al., 2008), and choreographic requirements (Bronner and Ojofeitimi, 2011).

**FIGURE 1:**
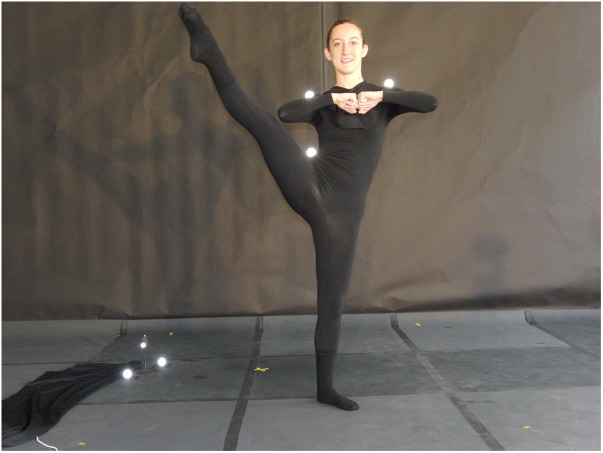
Developpé ending position. Written informed consent was obtained from the model for the publication of this image.

Increasing hip joint ROM is especially challenging and may result in loading the sacroiliac joint and lumbar spine (DeMann, 1997; Bronner and Ojofeitimi, 2011) if not done correctly (DeMann, 1997; Deckert et al., 2007; Gontijo et al., 2015). The developpé is a core ballet movement consisting of lifting the leg, performed routinely by dance students of all ages and levels of expertise. It can be performed in either a repeated (i.e., multiple repetitions) or static (i.e., holding the gesturing leg) manner and involves extreme hip flexion, abduction, and external rotation of the gesturing leg along with knee extension and ankle plantar-flexion (PF) (Figure 1; Agrippina, 1969; Bordier, 1975). Performing the developpé also entails controlling pelvic alignment (Martin et al., 1998; Gamboa et al., 2008; Bronner, 2012) and the appearance of “effortless” performance (Agrippina, 1969; Bordier, 1975; Wilson et al., 2004, 2007; Bronner and Ojofeitimi, 2006; Bronner and Shippen, 2015).

Hip ROM values during developpé performance vary among reports due to discrepancies regarding operational definitions (e.g., “hip ROM,” “gesture leg angle,” etc.), measurement protocols, and studied populations (Wilson et al., 2007; Krasnow et al., 2011): 73.1°± 12.9° in 11 female dance students and 74.4 ± 13.9° in 6 professional dancers, with no significant difference between the groups (Angioi et al., 2009); 99.9 ± 6.56–105.3°± 3.78 (right hip) in 20 dance students (Marshall and Wyon, 2012); 100 ± 16.96° in 20 female undergraduate dance students (Wyon et al., 2010); 116° ± 20° in 25 ballet dancers (Feipel et al., 2004); and 108°–130° in 6 professional ballet dancers (Martin et al., 1998).

The foot is often considered a focus of artistic attention (Agrippina, 1969; Hanrahan and Salmela, 1990; Laws, 2002) and serves as a reference point for the entire gesturing leg, with greater heights to which the leg (thus the foot) is lifted often indicating greater artistic quality and physicality. A study investigating developpé in 25 professional and semi-professional dancers found that the height of the leg depended more on hip flexibility than spinal contribution (Feipel et al., 2004).

Dynamic pelvic alignment (Franklin, 2012, 2014; Gontijo et al., 2015) and pelvic control (Gildea et al., 2015) are other important components for developpé performance (Holt et al., 2011), serving as a key element for whole-body axial postural alignment (Keller and West, 1995; DeMann, 1997; Wilson et al., 2004; Gontijo et al., 2015) and for facilitating efficient movement at the hip joint (Deckert et al., 2007). Correct pelvic dynamic alignment synchronized with the hip motion (known as “pelvi-femoral rhythm” (Bohannon et al., 1985; Murray et al., 2002) contributes to full hip ROM and efficient lower extremity motion (DeMann, 1997), especially in extreme positions such as developpé (Norkin and Levangie, 1992). Maintaining correct pelvic alignment (i.e., excessively tilted neither anteriorly nor posteriorly) is also important for achieving high aesthetics in dance movements (Agrippina, 1969; Bordier, 1975; Franklin, 2004, 2014; Holt et al., 2011) and may reduce the risk for low back, pelvic, and lower extremity injuries (DeMann, 1997; Deckert et al., 2007; Liederbach, 2010). A greater “hip-to-pelvis” ratio (i.e., greater hip vs. pelvic motion) is thus recommended for successful dance performance (Coker et al., 2015).

Improper pelvic alignment (e.g., excessive pelvic tilt), on the other hand, is undesired in dance and is a sign of lack of pelvic control (Deckert et al., 2007) that can increase the risk for injuries (Deckert et al., 2007; Gontijo et al., 2015). For example, excessive pelvic posterior tilt may serve as a compensation for insufficient hip ROM (Watelain et al., 2001) and may facilitate “tucking under” of the pelvis, thus over-loading the low back (Hagins, 2011) and impacting the dancer’s upright posture (Hanrahan, 1995; Franklin, 2014).

Previous literature suggested an increased pelvic motion among skilled dancers (about 7° more anterior pelvic tilt and 6.1° more lateral tilt toward the supporting leg) compared to novices (Wilson et al., 2004), and a positive correlation between the amount of pelvic motion and leg height (Wilson et al., 2007).

While full knee joint extension is also desired for developpé, knee hyper-extension in an effort to maximize performance is a suggested mechanism of knee injury in dance (Reid, 1988; Milan, 1994) and thus should be avoided.

The effectiveness of dance as well as other training approaches (e.g., stretching, strengthening) in increasing hip ROM among dance students is in debate (Bennell et al., 2001; Deighan, 2005; Steinberg et al., 2006; Wyon et al., 2006; Abraham et al., 2016). One study found that developpé ROM (as measured by hip ROM in 2-D) in a group of 20 dance students was increased by 17.6° (right hip) and 15.5° (left hip) following 8 sessions of whole body vibration (Marshall and Wyon, 2012). While it is widely agreed upon that dancers should learn to achieve a more neutral pelvic alignment (Deckert et al., 2007), technique classes alone did not improve pelvic alignment, whereas adding somatic training was found to lead to some improvement (Gamboian et al., 1999).

An understanding, therefore, of which training approaches for dancers can enhance their ROM, help maintain correct posture, and protect dancers from injuries is warranted. Such discoveries hold importance for the fields of performance enhancement, patho-mechanics of injuries (Bronner and Ojofeitimi, 2011), motor learning and control, and assessment of performance aesthetics (Flash et al., 1992; Flash and Sejnowski, 2001; Barliya et al., 2009, 2013; Bronner and Shippen, 2015).

Growing evidence supports the relevance of dance imagery [i.e. “the deliberate use of the senses to rehearse or envision a particular outcome mentally, in the absence of, or in combination with, overt physical movement” (Overby and Dunn, 2011)], for enhancing dance performance (Todd, 1937; Sweigard, 1978; Abraham et al., 2016, 2017; Pavlik and Nordin-Bates, 2016). Imagery can serve several goals, including directing attention; learning and improving dance skills, sequences, and timing; preventing injuries; and enhancing well-being (Hanrahan and Salmela, 1990; Minton, 1990; Fontin, 1993; Vaccaro, 1997; Overby et al., 1998; Vergeer and Hanrahan, 1998; Fish et al., 2004; Nordin and Cumming, 2006b; Enghauser, 2007; Allen and Wyon, 2008; Bolles and Chartfield, 2009; Overby and Dunn, 2011; Giron et al., 2012; Abraham et al., 2016, 2017; Pavlik and Nordin-Bates, 2016). Awareness of imagery by dance students and teachers has been suggested as an important component for developing the ability to better deal with ballet vocabulary and shapes (Johnson, 2011), and for supporting motor skill learning (Debarnot et al., 2014). Different types of motor tasks have been suggested to benefit differently from different imagery types (i.e., visual, kinesthetic) (Shenton et al., 2004; Giron et al., 2012; Lotze, 2013), with imagery being influenced by the individual’s motor habits (Willems et al., 2009). Specifically, dancers reported imaging dance skills and sequences, body-related images, and achieving goals (Nordin and Cumming, 2005).

Dancers’ imagery ability may improve following imagery training (Rodgers et al., 1991; Abraham et al., 2017). Several factors may determine dancers’ engagement, use, and benefit from imagery (Debarnot et al., 2014). These include previous experience with imagery, imagery ability, and learning strategy (Hall, 1985; Hall et al., 1985; Short et al., 2005; Nordin and Cumming, 2006a,b; Mulder et al., 2007; Bolles and Chartfield, 2009; Cumming and Williams, 2013; Abraham et al., 2017; Debarnot et al., 2014).

One study in 15 dancers found that a single, 30 min imagery session improved developpé performance (as measured by ankle height, with no plane of movement detailed) by ∼11–13 cm (no exact values or details regarding measurement method are given) (Hanrahan and Salmela, 1990). This improvement, however, was not followed by a subjective improvement (i.e., self-reported comfort level during task performance).

Among the existing imagery training approaches for dancers are motor imagery practice (MIP) (Pavlik and Nordin-Bates, 2016; Abraham et al., 2016, 2017), “Conditioning with Imagery” (Krasnow, 1997; Krasnow and Deveau, 2010; Krasnow et al., 2011), and Dynamic Neuro-Cognitive Imagery (DNI^TM^) (also known as “The Franklin Method^TM^” (Franklin, 2004, 2012, 2019; Heiland et al., 2012; Heiland and Rovetti, 2013).

DNI^TM^ is a codified, structured, imagery-based approach for movement and postural retraining, focusing on enhancing motor (e.g., range-of-motion, posture, and breathing) and non-motor (e.g., concentration, goal-setting, self-confidence) aspects of performance, and promoting optimal, safe dance practices. DNI^TM^ supports embodying functional human anatomy and biomechanics through movement and imagery, thus enhancing kinesthetic and proprioceptive imagery (Lotze, 2013), self-awareness, and perception. DNI^TM^ introduces and teaches students imagery and related techniques and tools (Franklin, 2004, 2014). These are used together with matching physical movements and exercises (Willems et al., 2009) for assuring embodiment. The contents of DNI^TM^ are tailored to direct the dancer’s attention to the cognitive, biomechanical, and sensory aspects of dance (Taylor and Taylor, 1995; Franklin, 2014, 2019; Beilock and Carr, 2001; Beilock et al., 2002), thus enriching their “toolbox.” A study investigating the effect of different DNI^TM^ images (i.e., visual, auditory, and kinesthetic) found all three to be beneficial in improving *plié arabesque* in 30 college dance students (Heiland et al., 2012). In another study, DNI^TM^ metaphorical images were found to be effective in improving jump height in 13 college dance students (Heiland and Rovetti, 2013). Recently, we have shown that training in DNI^TM^ improved motor and non-motor aspects of performance and pelvic schema in people with Parkinson’s disease (Abraham et al., 2018, 2019).

Yet gaps exist between research on imagery (Dickstein and Deutsch, 2007; Guillot and Collet, 2008; Bovend’eerdt et al., 2012) and the reality of imagery use and instruction (Overby et al., 1998; Nordin and Cumming, 2006b; Bolles and Chartfield, 2009), which is rarely formal but rather anecdotal during classes and rehearsals and greatly dependent on the teacher’s or choreographer’s background, expertise, and training (Nordin and Cumming, 2005, 2006a,b, 2007). In fact, few published studies have investigated the effect of the delivery of imagery training for dance students on motor and non-motor (e.g., self-confidence, concentration, self-reported levels of difficulty and proficiency, etc.) (Cumming and Williams, 2013) aspects of dance performance, and use of imagery during dance movements (Hanrahan and Salmela, 1990; Pavlik and Nordin-Bates, 2016).

Given growing interest in imagery as a complementary practice that integrates well with dance training (Adams et al., 2012; Batson and Wilson, 2014) and the promising evidence on this topic, there is a need to study the implementation and effects of specific, designated imagery training on dance performance, with the goal of facilitating structured imagery training in dance (Nordin and Cumming, 2006b), with detailed, well-established protocols (Overby and Dunn, 2011; Cooley et al., 2013; Pavlik and Nordin-Bates, 2016).

The aims of the present study were the following: (1) to describe imagery characteristics and preferred learning strategies in university-level dance students; (2) to investigate the effect of an intensive, 3 day DNI^TM^ training on participants’ imagery characteristics, developpé performance and kinematics, and self-reported perceptions; and (3) to investigate relationships between imagery characteristics and developpé performance following the intervention.

We hypothesized the following: (1) imagery ability and use, ankle height, pelvic and hip ROM, and self-reported level of proficiency and amount of improvement will improve following the intervention, level of difficulty will decrease, and knee and ankle ROM will not change following the intervention; and (2) baseline imagery scores and gains in imagery scores will be both positively correlated with amount of improvement in developpé performance.

## Materials and Methods

This study was carried out in accordance with the recommendations of the University of Georgia (Athens, GA, United States) Institutional Review Board, which approved the protocol. All subjects gave written informed consent before study commencement, in accordance with the Declaration of Helsinki. Inclusion criteria were active dance students at the university dance department, and 18 years of age and older. Exclusion criteria were any current musculoskeletal or other injuries limiting regular dance training routine. The study employed a within-subjects, double baseline repeated measures design with 3 time-points measurements: two pre-intervention measurements conducted 1 week (herein referred to as “pre 1”) and 48–72 h (herein referred to as “pre 2”) prior to intervention and a post-intervention measurement conducted 48–72 h after the intervention ended (herein referred to as “post”).

### Participants

A convenience sample of 34 university-level dance students (32 females, 2 males) from the University of Georgia dance department participated in the study. When relevant, participation in the study was offered to students as an additional option in fulfillment of an existing course assignment, equivalent in time, nature and effort, between which the students were free to choose. Participants’ demographics are detailed in Table 1.

**Table 1:** Participants’ demographics.

Gender	Combined (*n* = 34)	Females (*n* = 32)	Males (*n* = 2)
Age (years)	19.70 (1.57)	19.68 (1.60)	20.00 (1.41)
Height (cm)	165.63 (6.78)	164.68 (5.73)	180.75 (3.88)
Weight (kg)	59.61 (7.11)	59.11 (7.03)	67.60 (1.13)
Lower extremity length^†^ (cm)	83.64 (5.54)	83.59 (5.68)	84.50 (3.53)
**Highest Dancing Standard (n (%))**		
Recreational	1 (2.9%)	1 (3.1%)	
Increased interest	1 (2.9%)	1 (3.1%)	
Committed	11 (32.4%)	10 (31.3)	
Full-time student	12 (35.3%)	11 (34.4%)	1 (50%)
Advanced student	7 (20.6%)	7 (21.9%)	1 (50%)
Stronger leg (right:left) (n (%))	23 : 5 (82.2%:17.8%)	22 : 4 (84.6%:15.4%)	1 : 1 (50%:50%)
Balancing leg (right:left) (n (%))	8 : 19 (29.6%:70.4%)	7 : 18 (28%:72%)	1 : 1 (50%:50%)
Dancing experience (years)	13.61 (4.83)	14.15 (4.43)	5.00 (1.41)
Age first began to dance (years)	5.86 (4.90)	5.26 (4.38)	15.50 (0.70)
**Main Dance Type (n (%))**		
Ballet	20 (58.8)	20 (62.5)	
Contemporary	11 (32.4)	10 (31.3)	1 (50)
Hip-hop	1 (2.9)	1 (3.1)	1 (50)
Ballroom	1 (2.9)	1 (3.1)	
Jazz	1 (2.9)	20 (62.5)	
Previous experience with imagery (yes/no) (n (%))	9:24 (27.3%:72.7%)	9:22 (29%:71%)	0:2 (0%:100%)


*Values are means (SD) unless otherwise noted.*

*^†^Distance from greater trochanter to lateral malleolus.*

### The Developpé Tasks

Developpé was performed with the dancer standing in a starting position (i.e., 1st position, in which the lower extremities are externally rotated and heels touching), then drawing the gesturing (right) foot up to the knee of the supporting (left) leg into passé (hip flexion-abduction, knee flexion, and ankle PF of the gesturing leg), straightening the leg to an “open position” (i.e., gesturing knee extension with ankle PF) to maximal height while maintaining correct form (e.g., upright posture, correct supporting leg alignment, etc.) (Figure 1), then lowering the gesturing leg straight down to the floor (gesturing hip adduction-extension), and closing the gesturing leg back into starting position (hip adduction) (Agrippina, 1969; Bordier, 1975). Upper extremities were kept in a modification of port de bras 1st position (fists touching in front of sternum and elbows kept slightly up) throughout performance of all three tasks. Participants performed three randomly ordered developpé tasks (see below), representing the scope and requirements of developpé practice (i.e., duration and number of repetitions) in university-level ballet classes. In addition, such motor tasks address the kinematic differences existing between static and repeated performance of dance movements (Abraham et al., 2016, 2017).

The three developpé tasks were the following:

(1) “Repeat” – four consecutive repetitions of developpé. The exact instruction was, “Perform 4 repetitions of developpé in a row as high as you can and at your own tempo, while maintaining your form.”
(2) “Static” – a continuous developpé for 8 consecutive seconds. The exact instruction was, “Perform a developpé and hold it as high as you can for 8 seconds, according to my counts, while maintaining your form.”
(3) “Single” – a single repetition of developpé. The exact instruction was, “Perform one repetition of developpé as high as you can while maintaining your form.”

Participants were instructed to perform the “repeat” and “single” tasks at their preferered tempo (i.e., the tempo they were used to while practicing developpé in ballet classes).

### Dynamic Neuro-Cognitive Imagery (DNI^TM^) Intervention

The goal of the DNI^TM^ intervention was to equip participants with imagery-related knowledge and skills for enhancing their developpé performance while focusing on correct, safe, and mindful movement. The intervention did not include exact performance of the three tasks, as performed during the measurement sessions. This was done deliberately with the goal of focusing on the effect of DNI^TM^ elements within performance rather than physical-actual training effects.

The specific objectives of the intervention were to provide participants with the following elements:

(1) Introduction to imagery (Wondrusch and Schuster-Amft, 2013) and foundations of imagery skills and use in dance training and performance (Franklin, 2012, 2014).
(2) Understanding and embodiment of correct alignment and use of the pelvis and hip joint throughout developpé performance (e.g., balancing the pelvis on the heads of femurs (Franklin, 2004, 2012, 2014; Laws, 2002; Deckert et al., 2007).
(3) Improving hip joint flexibility and increasing hip flexion and abduction ROM.
(4) Enhancing developpé performance (i.e., increased ankle height).

The DNI^TM^ sessions focused on embodying correct pelvic and hip biomechanics, releasing muscular tension around the pelvis and hip, and facilitating efficient work of relevant muscles (e.g., ilio-psoas and glutei muscles), all through imagery and specifically in relation to developpé. Anatomical plastic models were used to demonstrate correct pelvic and hip “bone rhythms” (Franklin, 2004, 2012, 2014; Heiland et al., 2012) during developpé as well as developpé-specific DNI^TM^ images and metaphors (Table 2).

**Table 2:** Sections and examples of imagery exercises of the dynamic neurocognitive imagery session.

**Introduction to imagery**
• Introduction to imagery training as an effective method for dance performance enhancement (e.g., mechanisms of effect) • Sub-types of imagery (e.g., kinesthetic, visual) and perspectives (1st vs. 3rd person) • Engagement into imagery, self-awareness, and concentration • Selected DNI^TM^ tools and strategies: e.g., positive self-talk (Theodorakis et al., 2000), anatomical, metaphorical, and motivational imagery (Franklin, 2004, 2012; 2014; Hanrahan and Vergeer, 2000)
**Pelvic alignment and control**
• Pelvic structure, function, and motion • Pelvic osteo- and arthro-kinematics during developpé performance (Known as DNI^TM^ “bone rhythms”) • Anatomical imagery: differentiation between right and left pelvic halves (i.e., innominate bones) • Metaphorical imagery: “Pelvic half as a wheel” for addressing anterior and posterior pelvic tilt (Franklin, 2004)
**Hip Joint**
• Identification and embodiment of hip joint location • Femoral head and shaft osteo-kinematics (i.e., roll/spin accessory movements) • “Pelvi-femoral rhythm” (counter-rotations) (Known as DNI^TM^ “bone rhythms”) • Muscular tension release (e.g., glutei, deep external rotators) while lifting the thigh
**Spine**
• Anatomical imagery: spine supported on top of the pelvis, correct spinal alignment • Biomechanical imagery: central axis for maintaining upright posture • Metaphorical imagery: upright, subtle spine (“spine as a spring,” “spine as a rocket”) (Franklin, 2004, 2012, 2014)
**Developpé-specific**
Metaphorical imagery: • Balancing on supporting leg and foot: “supporting foot is sinking in sand,” “supporting foot sending a tree’s roots to the ground”) (Franklin, 2004; 2012, 2014) • Opposition between gesturing and supporting legs: “opening fan” • Smooth, effortless leg rise: “Helium balloon lifting the leg”


*The DNI^*TM*^ intervention is comprised of several segments that deal with introducing the participant to the DNI^*TM*^ technique, pelvic alignment and control, hip joint and spinal visualization and metaphorical images that accompany the practice. Listed in this table are some examples of images provided to the participant during classes. For this study, developpe specific imagery was developed and practiced.*

The intervention consisted of 3 × 1.5 h group sessions over 3 consecutive days over the first third of the academic semester (weeks 4–5). Sessions were offered twice a day to accommodate participants’ schedules and took place in the same dance studio where participants attended classes regularly. The intervention was delivered by two DNI^TM^ master educators with extensive experience in teaching imagery for dancers. Each session included the following components: (1) DNI^TM^ and physical warm-up (10 min); (2) DNI^TM^ techniques and exercises for improving developpé biomechanics, balance, ROM, and body alignment (70 min); and (3) cool-down (10 min) which included answering questions, relaxation, and “take-home” messages. The 1st session included an introduction to imagery and DNI^TM^ for familiarizing participants with this training approach, based on teachers’ experience and previous literature (Bovend’eerdt et al., 2012; Wondrusch and Schuster-Amft, 2013). The 3rd session included individual work with the DNI^TM^ educators for addressing specific concerns and challenges for each participant as well as for fine-tuning the DNI^TM^ contents and use. Examples of the intervention’s contents are detailed in Table 2.

### Measurement Protocol

Participants were measured at three different time-points: one week prior to the intervention (“pre 1”), 72–48 h prior to the intervention (“pre 2”), and 48–72 h after the intervention ended (“post”). All three measurements were identical in protocol. Participants wore a black-colored unitard to maximize reflective markers’ contrast. Fourteen markers were attached using a double-sided adhesive tape to the following anatomical landmarks: acromion (x2), C_7_ and T_12_ vertebrae, posterior superior iliac spines (“PSIS” marker; x2), top of iliac crest (“iliac crest” marker), greater trochanter (“hip” marker), lateral knee joint line (“knee” marker; x2), mid-shank (x2), lateral malleolus (“ankle” marker), and 5th metatarsal-phalangeal joint (“foot” marker).

Participants were asked to warm up using their preferred routine (i.e., stretching, aerobic exercises, etc.) for 5–7 min prior to the commencement of data collection. Participants stood in their preferred 1st position (in terms of amount of hips external rotation) with a sign on the floor marking the heels’ touching point. Using participants’ preferred amount of hip external rotation ROM was chosen to allow accommodating for physical diversity among dancers (Gamboian et al., 1999; Champion and Chatfield, 2008). Upper extremities were kept in the modified port de bras 1st position throughout performance of all three tasks in order to diminish the impact of the arms in aiding balance and to minimize interference with the reflective markers. The developpé movement was performed using the right lower extremity as the gesturing leg. Neither a ballet barre nor a mirror was available during measurements. Loud counting of the repetitions (for the “repeat” task) or seconds (for the “static” task) was provided to participants by the researcher (AA). Duration of developpé during “static” task was measured using a digital watch. No additional instructions/cues were provided. In case of loss of balance (i.e., the gesturing leg touching the floor) before task completion, the measurement was stopped and marked as “incomplete” for data analysis purposes.

After each task’s completion over the three measurements, participants were asked to rate on a 1–7 Likert scale their self-perceived levels of proficiency (“How well did you perform on a scale of 1–7, with 1 representing “not well at all” and 7 representing “best,”), and difficulty (“How would you rate the difficulty of this performance on a scale of 1–7, with 1 representing “not difficult at all” and 7 representing “very difficult”). For “pre 2” and “post” measurements, participants were also asked to rate their self-perceived amount (in percent) of improvement in task’s performance in comparison to the previous measurement (“in comparison to previous assessment, by how much did you improve your performance, on a scale from 0 to 100%?”). Following previous recommendations for assessing imagery use in dancers (Pavlik and Nordin-Bates, 2016), during the “post” measurement, participants were asked immediately after each task’s completion whether they used any of the DNI^TM^ images/metaphors. Answers were recorded in writing by a research assistant.

### Data Collection and Processing

Demographic data, including a laterality questionnaire (Mertz and Docherty, 2012), were collected at “pre 1.” Kinematic data were collected at 120 Hz using two digital cameras (Casio© Exilim *FH20*) positioned 6 m posteriorly and 60° postero-laterally to the participant’s right lower extremity. Collecting data from the right leg only is in keeping with previous dance performance kinematics investigations (Bennell et al., 2001; Wilson et al., 2004; Gerbino et al., 2007; Bronner and Ojofeitimi, 2011; Gontijo et al., 2015; Abraham et al., 2016, 2017). System calibration was performed at the beginning of each measurement session using a 32-point calibration frame (Peak Motus©, Vicon Motion Systems, Inc., CO, United States). For “repeat” and “single” tasks, “ending position” was determined by peaks of developpé performance, determined by maximal vertical linear displacements of the “ankle” marker (i.e., the maximal height of the ankle from the floor). For the “static” task, the beginning of the plateau phase followed by 8 consecutive seconds (i.e., plateau phase) was determined and extracted using a MATLAB© software (Version *R2011a*) code. When in question, peak of performance and beginning of plateau phase were verified through videos. All kinematic variables (see “outcome measures”) were determined based on time-points of peaks of performance (for “repeat” and “single” tasks) and beginning of plateau (for “static” task). Kinematic raw data were processed using the APAS© (Ariel Performance Analysis System) software (version 13.3.0.1) by Ariel Dynamics© (Abraham et al., 2016, 2017). Quintic Spline (Vaughan, 1982) was used for filtering data. Further processing was made using a MATLAB© software *(*Version R2016a) code. Means and standard deviations (SD) for the 4 repetitions and 8-scond plateau phase were then calculated and used for statistical analysis (see “Kinematic Dependent Variables” paragraph).

Mental imagery and preferred learning strategy questionnaires were administered at “pre 1” and “post,” allowing calculating improvements/gains following the intervention.

For data analysis purposes, an inclusion criteria of 100% participation in the intervention (i.e., all 3 sessions) and attendance in the “post” measurement were established.

### Outcome Measures

Retention rate was defined as the percentage of participants who completed both the intervention and the “post” measurement out of those completed the “pre 1” measurement. Adherence to intervention was established using percentage of the DNI^TM^ sessions (out of all 3 sessions) attended by participants.

#### Mental Imagery

(1) Vividness of Movement Imagery Questionnaire 2 (VMIQ-2) (Roberts et al., 2008; Callow and Roberts, 2010) – a 36-item questionnaire for assessing vividness of movement imagery. The questionnaire consists of 3 categories (i.e., external visual, internal visual, and kinaesthetic, with 12 identical questions for each category). Answers were on a Likert scale ranging from 1 (“perfectly clear and vivid as normal vision/feel of movement”) to 5 (“no image at all, you only know that you are thinking of the skill”), with lower values representing better vividness. The VMIQ-2 has demonstrated acceptable factorial validity, construct validity, and concurrent validity (Roberts et al., 2008). A different version of VMIQ-2 was previously described for assessing imagery ability in dancers (Di Corrado et al., 2014).

(2) Dance Imagery Questionnaire (DIQ) (Nordin and Cumming, 2006a) – a 16-item questionnaire for assessing the frequency with which dancers engage in 4 imagery types (i.e., the content of the image): Technique (i.e., skill and movement sequences), Goals (e.g., working toward and reaching dance-related goals), Role and Movement Quality (e.g., images of an artistic nature), and Mastery (e.g., staying focused, dealing with difficulty, planning, and control of anxiety) (Nordin and Cumming, 2008). Answers are on a Likert scale ranging from 1 (“never”) to 7 (“very often”), with higher values representing greater frequency. The DIQ has adequate psychometric properties in dancers, including a cross-validated factor structure, adequate test-retest reliability, and Cronbach’s alpha values ranging from 0.81 to 0.87 (Nordin and Cumming, 2006a).

#### Preferred Learning Strategy

(1) The Visual-Aural-Read/Write-Kinesthetic (VARK) Questionnaire (^©^Copyright Version 7.8 (2014) held by VARK Learn Limited, Christchurch, New Zealand) (Leite et al., 2010; Urval et al., 2014) – a 16- multiple-choice items questionnaire designed to assess four learning style/sensory modalities preferences [i.e., visual (V), aural (A), read/write (R), and kinesthetic (K)]. For all 16 questions, participants can choose one or more answers with the four options corresponding to the four learning modalities. First modality preference was determined based on the highest of the four scores. Uni- or multi-modal preference was calculated using the “stepping stone” scoring algorithm (Fleming and Bonwell, 2001) to determine whether the participant is unimodal (i.e., V, A, R, K) or multi-modal (MM). A multi-modal preference occurs when a person has a strong preference for 2 or more of the VARK modalities (Fleming and Bonwell, 2001). The questionnaire was validated for use with athletes (Boyde et al., 2009), was used with dancers (Heiland et al., 2012), and was specifically recommended for use in college dancers (Heiland et al., 2012). The Copyright permission for the use of the VARK inventory was send via email (November 2016) and approval granted for the paper version only.

#### Kinematic

(1) Ankle Height – the height (in Cm) of the right “ankle” marker from the floor at ending position. Ankle height has been recommended as a parameter for measuring dance performance (Welsh, 2003) and used for assessing developpé (Hanrahan and Salmela, 1990) and other dance movements (Grossman and Wilmerding, 2000).

(2) Ankle Plantar-Flexion (PF)– the ankle PF angle (in Degrees) at ending position. Ankle angle was formed by the “foot” segment (represented by the line connecting the “foot” marker and the “ankle” marker) and the “shank” segment (represented by the line connecting the “ankle” marker and the “knee” marker).

(3) Hip Flexion – the hip motion (in Degrees) in the sagittal plane. Hip angle was formed by the “crest” segment (represented by the line connecting the “iliac crest” marker and the “hip” marker) and the “thigh” segment (represented by the line connecting the “hip” marker and the “knee” marker).

(4) Hip Abduction – the amount of hip motion (in Degrees) in the frontal plane. Hip angle was formed by the “crest” segment (represented by the line connecting the “iliac crest” marker and the “hip” marker) and the “thigh” segment (represented by the line connecting the “hip” marker and the “knee” marker).

(5) Knee Extension – the knee angle (in Degrees) as formed by the “thigh” segment (represented by the line connecting the “hip” marker and the “knee” marker) and the “shin” segment (the line connecting the “knee” marker and the “ankle” marker).

(6) Innominate Posterior Tilt – the right innominate poterior motion angle (in Degrees) in the sagittal plane as measured by the “innominate” segment (the line connecting the“iliac crest” marker and the “PSIS” marker).

(7) Pelvic Hiking (Lateral Tilt) – the pelvic motion angle (in Degrees) in the frontal plane. Angle was measured using the “pelvis” segment ( the line connecting the left and right “PSIS” markers).

### Statistical Analysis

Descriptive statistics were used to calculate participants’ characteristics and demographics. A repeated measures analysis of variance (ANOVA) was used to calculate the within-group change in kinematic and self-reported outcome measures between the three measurements. Paired-samples *t*-test were used for comparing “pre 1” and “post” questionnaires. Two-tailed hypotheses were used with *p* < 0.05 regarded as significant. Range and 95% confidence intervals (95%CI) were calculated when appropriate. Effect sizes were calculated using partial Eta Squared (*η*_p_*^2^*). *Post hoc* analyses using Bonferroni corrections were used. Correlations were calculated using Pearson’s Correlation Coefficients. All analyses were conducted using SPSS^®^ software (version 19.0, SPSS Inc., Chicago, IL, United States). One participant dropped from the study before attending the “post” measurement for unknown reasons, and was thus excluded from data analysis.

## Results

Retention rate was 97.05% (33 out of 34 participants completed the intervention and “post” measurement). Adherence rate was 100%, with all 33 participants attending the 3 DNI^TM^ sessions (out of 3 offered). All 33 participants successfully completed the 3 developpé tasks during 3 measurement sessions.

**FIGURE 2:**
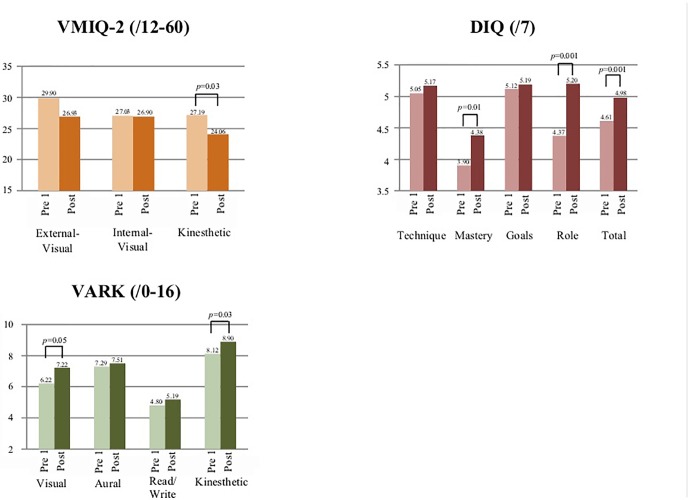
Visual representation of participants’ mental imagery characteristics and preferred learning modalities over time.

Imagery characteristics scores are presented in Table 3 and Figure 2. No significant differences (*p* > 0.05) were detected at “pre 1” between the three VMIQ-2 scores. Statistically significant positive correlations were detected between scores of all three categories (Table 4).

**Table 3:** Participants’ mental imagery characteristics and preferred learning modalities over time^†^.

	Pre 1	Post	t {95%CI}	*p*
**VMIQ-2 (/12–60)**		
External-visual	29.90 ± 12.65 [12.00–52.00]	26.93 ± 9.42 [12.00–46.00]	1.93 {-0.16–6.09}	0.06
Internal-visual	27.03 ± 9.42 [12.00–50.00]	26.90 ± 9.43 [12.00–41.00]	0.10 {-2.35–2.61}	0.91
Kinesthetic	27.19 ± 9.60 [13.00–52.00]	24.06 ± 8.31 [12.00–41.00]	2.24 {0.28–5.97}	0.03^∗^
**DIQ (/7)**		
Technique	5.05 ± 0.95 [2.25–7.00]	5.17 ± 0.83 [3.50-6.75]	0.80 {-0.19–0.44}	0.43
Mastery	3.90 ± 1.14 [1.25–6.00]	4.38 ± 1.10 [2.50–6.50]	2.62 {0.10–0.84}	0.01^∗∗^
Goals	5.12 ± 1.10 [1.75–7.00]	5.19 ± 0.97 [3.25–7.00]	0.45 {	0.65
Role	4.37 ± 1.17 [2.25–6.75]	5.20 ± 0.85 [3.75–6.50]	4.79 {0.47–1.17}	0.001^∗∗^
Total	4.61 ± 0.85 [2.38–6.38]	4.98 ± 0.82 [3.63–6.63]	3.52 {0.15–0.58}	0.001^∗∗^
**VARK (/0–16)**		
Visual	6.22 ± 2.82 [1–12]	7.22 ± 4.05 [0–15]	2.03 {0.00–2.00}	0.05^∗^
Aural	7.29 ± 2.90 [2–14]	7.51 ± 3.62 [1–14]	0.44 {-0.081–1.26}	0.66
Read/Write	4.80 ± 2.35 [0–10]	5.19 ± 3.08 [1–12]	0.84 {-0.59–1.36}	0.42
Kinesthetic	8.12 ± 2.76 [0–13]	8.90 ± 3.20 [0–14]	2.27 {0.08–1.46}	0.03^∗^
Uni:Multi-modal	11:22 (33.33%:66.66%)	12:19 (38.70%:61.30%)	0.20^a^	0.65
**VARK 1^ST^ Preference [n, (%)]**		
Visual	5 (19.23%)	8 (28.57%)		
Aural	10 (38.46%)	5 (17.86%)		
Read–write	2 (7.70%)	3 (10.71%)		
Kinesthetic	9 (34.61%)	12 (42.86%)		


*^†^Differences were calculated using paired-samples t-test, unless otherwise specified.*

*Values are M (SD) [range] unless otherwise indicated.*

^∗^*p < 0.05.*

^∗∗^*p < 0.01.*

*^*a*^Chi Square Test of Independence.*

**Table 4:** Correlations between VMIQ-2 imagery modalities scores at baseline.

	External-visual	Internal-visual
Internal-visual	0.741^∗∗^	-
Kinesthetic	0.342^∗^	0.615^∗∗^


*Values are Pearson’s correlation coefficients.^∗^p < 0.05.^∗^*^∗^*p < 0.01.*

The DIQ Goals score was the highest and significantly higher than the Mastery (*p* < 0.01) and Role (*p* < 0.05) scores.

The VARK scores are presented in Table 3 and Figure 2. Kinesthetic score was the highest and was significantly higher than the Visual (*p* < 0.01) and Read/Write (*p* < 0.01) sub-scores but not from the Aural score (*p* > 0.05).

**FIGURE 3:**
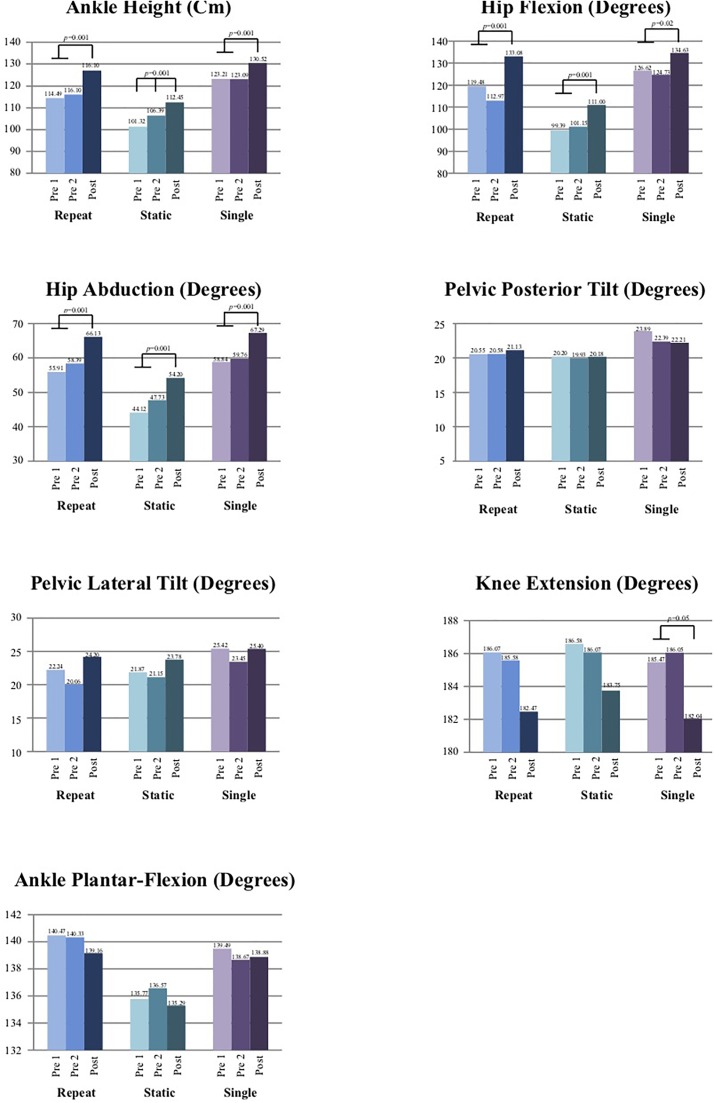
Visual representation of performance and kinematics results.

Performance and kinematic outcome measures for the three measurements are presented in Table 5 and Figure 3. Developpé performance (as measured by ankle height) improved significantly (*p* < 0.01) between “pre 2” and “post” for the “repeat” and “single” tasks, with no such improvements between “pre 1” and “pre 2”. For the “static” task, ankle height improved significantly (*p* < 0.01) over time (from “pre 1” to “pre 2” and from “pre 2” to “post”). Hip flexion and abduction ROM in all three tasks improved significantly (*p* < 0.01) between “pre 2” and “post” with no improvements between “pre 1” and “pre 2”. Pelvic posterior and lateral tilts did not change significantly between the three time points in all three tasks. Knee extension for the “single” task decreased significantly following the intervention while exhibiting less hyper-extension.

**Table 5:** Developpé performance and kinematic outcome measures over time.

	Pre 1	Pre 2	Post	*F*_(2,__64)_	*p*	*η*_P_^2^
**Ankle Height (Cm)**		
“Repeat”	114.49 ± 20.78	116.10 ± 18.36	127.10 ± 17.94	32.99	0.001^∗∗^	0.508
	[107.12–121.86]	[109.59–122.61]	[120.73–133.46]			
“Static”	101.32 ± 21.90	106.39 ± 21.92	112.45 ± 21.44	31.43	0.001^†^	0.496
	[93.55–109.09]	[98.62–114.16]	[104.84–120.05]			
“Single”	123.21 ± 19.14	123.09 ± 17.85	130.52 ± 17.80	18.20	0.001^∗∗^	0.363
	[117.56–128.85]	[117.83–128.36]	[125.27–135.77]			
**Hip Flexion (Degrees)**		
“Repeat”	119.48 ± 24.99	112.97 ± 37.72	133.08 ± 19.14	5.78	0.001^∗∗^	0.153
	[110.62–128.34]	[99.59–126.35]	[126.29–139.87]			
“Static”	99.39 ± 26.88	101.15 ± 29.97	111.00 ± 25.08	7.53	0.001^∗∗^	0.191
	[89.86–108.92]	[90.52–11.78]	[102.34–120.14]			
“Single”	126.62 ± 26.27	124.73 ± 23.94	134.63 ± 19.19	3.91	0.02^∗^	0.109
	[117.30–135.94]	[116.24–133.22]	[127.83–141.44]			
**Hip Abduction (Degrees)**		
“Repeat”	55.91 ± 12.10	58.39 ± 11.83	66.13 ± 10.73	14.58	0.00^∗∗^	0.313
	[51.62–60.21]	[54.20–62.59]	[62.32–69.93]			
“Static”	44.12 ± 14.36	47.73 ± 14.91	54.20 ± 13.53	18.96	0.00^∗∗^	0.372
	[39.02–49.21]	[42.44–53.02]	[49.41–59.00]			
“Single”	58.84 ± 13.31	59.76 ± 12.98	67.29 ± 12.14	10.09	0.00^∗∗^	0.240
	[54.12–63.56]	[55.15–64.36]	[62.98–71.59]			
**Pelvic Posterior Tilt (Degrees)**		
“Repeat”	20.55 ± 6.68	20.58 ± 7.97	21.13 ± 5.72	0.19	0.82	0.006
	[18.18–22.92]	[17.75–23.41]	[19.10–23.16]			
“Static”	20.20 ± 6.48	19.93 ± 8.14	20.18 ± 5.68	0.05	0.94	0.002
	[17.91–22.50]	[17.04–22.82]	[18.16–22.20]			
“Single”	23.89 ± 6.83	22.39 ± 8.07	22.21 ± 5.76	1.53	0.22	0.046
	[21.47–26.32]	[19.53–25.26]	[20.17–24.25]			
**Pelvic Lateral Tilt (Degrees)**		
“Repeat”	22.24 ± 8.05	20.06 ± 8.16	24.20 ± 8.41	6.51	0.43	0.169
	[19.39–25.10]	[17.17–22.96]	[21.22–27.19]			
“Static”	21.87 ± 7.72	21.15 ± 9.03	23.78 ± 8.52	4.00	0.20	0.111
	[19.13–24.61]	[17.95–24.36]	[20.76–26.80]			
“Single”	25.42 ± 8.48	23.45 ± 8.55	25.40 ± 9.00	2.03	0.14	0.060
	[22.41–28.43]	[20.42–26.48]	[22.21–28.60]			
**Knee Extension (Degrees)**		
“Repeat”	186.07 ± 17.73	185.58 ± 20.70	182.47 ± 14.70	2.13	0.12	0.063
	[179.78–192–36]	[178.24–192.92]	[177.25–187.69]			
“Static”	186.58 ± 10.42	186.07 ± 11.39	183.75 ± 9.22	2.35	0.10	0.068
	[182.88–190.27]	[182.03–190.12]	[180.48–187.03]			
“Single”	185.47 ± 14.56	186.05 ± 18.67	182.04 ± 15.92	3.08	0.05^∗^	0.088
	[180.30–190.63]	[179.43–192.67]	[176.39–187.69]			
**Ankle Plantar–Flexion (Degrees)**		
“Repeat”	140.47 ± 8.71	140.33 ± 9.99	139.16 ± 9.69	0.79	0.45	0.024
	[137.38–143.56]	[136.79–143.87]	[135.72–142.60]			
“Static”	135.77 ± 9.75	136.57 ± 11.73	135.29 ± 10.01	0.67	0.51	0.021
	[132.31–139.23]	[132.41–140.73]	[131.74–138.85]			
“Single”	139.49 ± 9.37	138.67 ± 12.47	138.88 ± 9.60	0.18	0.83	0.006
	[136.17–142.81]	[134.25–143.10]	[135.47–142.29]			


*Values are M (SD) [95% CI] unless otherwise indicated.*

*^∗^p < 0.05.*

*^∗∗^p < 0.01.*

*^†^Significant differences between all 3 measurements.*

Participants’ self-reported data are presented in Table 6. Self-reported level of proficiency improved significantly at “post” measurement for the “repeat” task (*p* < 0.01). Self-reported level of difficulty in the “repeat” task decreased significantly following the intervention and self-reported amount of improvement increased significantly following the intervention in all three tasks.

**Table 6:** Self–reported outcome measures associated with developpé performance.

	Pre 1	Pre 2	Post	*F*_(2,__64)_	*p*	*η*_P_^2^
**Level of Proficiency (/7)**		
“Repeat”	4.39 ± 0.87	4.57 ± 0.83	5.16 ± 0.80	16.24	0.00^∗∗^	0.337
“Static”	4.27 ± 1.19	4.39 ± 0.90	4.72 ± 1.11	2.66	0.07	0.077
“Single”	4.62 ± 1.03	4.68 ± 1.10	5.00 ± 0.94	2.92	0.06	0.084
**Level of Difficulty^¶^ (/7)**		
“Repeat”	3.89 ± 1.27	3.60 ± 1.02	2.96 ± 0.91	14.57	0.00^∗∗^	0.313
“Static”	5.04 ± 0.95	4.71 ± 1.10	4.37 ± 1.19	7.91	0.00^†^	0.198
“Single”	3.37 ± 1.15	3.27 ± 1.11	2.87 ± 1.08	3.51	0.03^†^	0.099

	**Pre 2**	**Post**	**95% CI of Difference**		***t*_(32_*_)_***	***p***

**Amount of Improvement (%)**						
“Repeat”	13.63 ± 18.03	39.60 ± 23.75	25.96 (16.81–35.12)		5.77	0.00^∗∗^
“Static”	12.72 ± 17.41	32.03 ± 25.70	19.30 (11.70–26.90)		5.17	0.00^∗∗^
“Single”	10.06 ± 13.36	32.87 ± 25.80	22.81 (14.70–30.92)		5.73	0.00^∗∗^


*Values are means (SD) unless otherwise noted; 95% CI = 95% Confidence Intervals.****^¶^****Lower values represent less difficulty.*

^∗^*p < 0.05.*

^∗∗^*p < 0.01.*

*^†^Overall difference from “pre 1” to “post.”*

Correlations between VMIQ-2 and developpé performance (as measured by ankle height) baseline scores and gains (i.e., the “post-pre” difference) are presented in Table 7. Statistically significant negative correlations (*p* < 0.01) were detected between VMIQ-2 baseline scores and gains for each of the three modalities, suggesting that participants with better imagery ability (i.e., lower values) improved less (i.e., greater values) in their imagery ability following the intervention. None of the baseline VMIQ-2 scores was significantly correlated with gains in developpé performance in any of the 3 tasks. Gain in VMIQ-2 kinesthetic gain was statistically significantly negatively correlated with gains in developpé performance for the “repeat” (*p* < 0.01) and “single” (*p* < 0.05) tasks, suggesting that greater gains in kinesthetic imagery (i.e., lower values) were positively correlated with greater gain in developpé performance (i.e., greater values).

**Table 7:** Correlations between VMIQ–2 and developpé performance baseline scores and gains.

	“Repeat” gain	“Static” gain	“Single” gain	External visual gain	Internal visual gain	Kinesthetic gain
Baseline external visual	0.060	–0.102	–0.151	–0.668^∗∗^	–	–
Baseline internal visual	–0.052	–0.312	–0.215	–	–0.358^∗^	–
Baseline kinesthetic	0.166	–0.085	0.077	–	–	–0.560^∗∗^
External visual gain	–0.205	–0.181	0.063	–	0.213	0.215
Internal visual gain	–0.246	0.067	–0.217	–	–	0.532^∗∗^
Kinesthetic gain	–0.416^∗∗^	–0.211	–0.355^∗^	–	–	–


*Values are Pearson’s correlation coefficients.*

*^∗^p < 0.05.*

*^∗∗^p < 0.01.*

## Discussion

The current study implements previous recommendations in dance and imagery literature for within-subjects repeated measures designs (Gamboian et al., 1999; Deckert et al., 2007) and for conducting measurements in the dancers’ natural environment (i.e., a dance studio) rather than in a lab (Deckert et al., 2007), thus increasing the results’ validity and relevance.

The high retention and adherence rates (97.05% and 100%, respectively) are similar to those previously reported for a motor imagery intervention in adolescent dance students (96.00% and 93.04% for retention and adherence, respectively) (Abraham et al., 2017) and suggest that this intervention was of interest for the participants.

The VMIQ-2 baseline scores in the current study are similar to those of 240 low-level/recreational athletes [30.22 ± 10.76 (External-visual), 27.14 ± 11.31 (Internal-visual), and 28.10 ± 11.26 (Kinesthetic)] (Roberts et al., 2008) and to External-visual score (20.38–28.38) and Kinesthetic score (range: 19.38–21.75) in 24 elite female dancers (Coker et al., 2015). Given a lack of literature regarding minimum clinically important difference (Copay et al., 2007) in mental imagery measure, no additional insights are available at this point. The non-significant differences between the three VMIQ-2 baseline sub-scores (Figure 2 and Table 3) align with previous reports (Di Corrado et al., 2014) and support the notion that dance students have similar visual and kinesthetic imagery capabilities (Pavlik and Nordin-Bates, 2016), including using both internal and external pesepctives in tandem (Vergeer and Hanrahan, 1998). The finding of statistically significant positive correlations between the VMIQ-2 sub-scores (Table 4) further support this assumption and may also suggest some degree of similarity existing between these modalities (Callow et al., 2013): specifically, the correlation between Internal-visual and Kinesthetic modalities. The significant increase in VMIQ-2 Kinesthetic score following the intervention is of interest, given literature describing kinesthetic imagery as experienced less frequently by dancers and potentially more difficult/challenging to engage with (Lotze, 2013; Coker et al., 2015), and given some literature suggesting that dancers may have lesser kinesthetic imagery ability in comparison to the visual one (Nordin and Cumming, 2006a; Coker et al., 2015) Therefore, the significant increase in kinesthetic ability in the current study may imply a potential to improve this skill following DNI^TM^ training. Moreover, our results suggest that gains in kinesthetic imagery ability following the intervention may have played a role in the improvements noticed in developpé performance (Table 7), thus further emphasizing the importance of this imagery type (Lotze, 2013).

The DIQ baseline scores are somewhat similar to previous scores reported in a study of 250 dancers (*M* age = 23.82 ± 9.16) [4.69 ± 1.23 (Total), 5.08 ± 1.24 (Technique), 4.22 ± 1.34 (Mastery), 4.87 ± 1.46 (Goals), and 4.62 ± 1.36 (Role)] (Nordin and Cumming, 2006a) and in a study of 144 dancers [5.20 ± 0.94 (Technique), 4.52 ± 1.09 (Mastery), 4.82 ± 1.10 (Goals), and 4.63 ± 1.19 (Role)] (Nordin and Cumming, 2008). The finding of the Goals sub-score being the highest may illuminate the importance and relevance this type of imagery might have for university-level dance students.

The finding of 66% of the participants presenting a multi-modal preferred learning strategy (Table 3) may align with the above-described multi-modality imagery ability. This may support the notion that dancers tend to use more multi-sensory imagery as they become more experienced (Nordin and Cumming, 2006b). The improvements noticed following the intervention in the DIQ Mastery, Role, and Total scores suggest a positive effect of the DNI^TM^ intervention on non-motor aspects of performance, including performance anxiety, concentration, and emotional states, and potentially creativity in regards to metaphors creation (Nordin and Cumming, 2006a). Specifically, the improvement in the Mastery sub-score is noteworthy in that it was reported to be the least utilized by dancers (Nordin and Cumming, 2006a, 2008) and was associated with higher self-confidence and lower anxiety in dancers (Fish et al., 2004; Monsma and Overby, 2004; Nordin and Cumming, 2006a, 2008).

The statistically significant increases in the VARK visual and kinesthetic scores following the DNI^TM^ intervention could be explained by these two imagery strategies being rooted within and widely-used in DNI^TM^, thus potentially pointing at a relationship between imagery and learning strategies in dancers (Bolles and Chartfield, 2009).

The DNI^TM^ intervention resulted in significant improvements in developpé performance of the “repeat” and “single” tasks. In addition, the intervention increased hip flexion and abduction ROM in all three tasks. These gains were accompanied by significant increase in self-reported level of proficiency and a significant reduction in level of difficulty (“repeat”) and significant gains in amount of improvement (all three tasks). These results suggest that gains in both objective and self-reported measures are possible following imagery training, unlike previous reports showing that objective improvements were not accompanied by self-reported ones (Hanrahan and Salmela, 1990). In addition, these findings are especially noteworthy, given the short time period (i.e., 3 days) and participants’ familiarity and experience with the tasks. The former is important given the prolonged dance training thought to be required for achieving motor gains (e.g., pelvic motor control) (Bronner, 2012). Furthermore, this may suggest that the particpants, despite being experienced with developpé, were successful at developping new patterns of thought regarding developpé through using imagery (Debarnot et al., 2014).

The improvements of 11 cm (9.48%) and 7 cm (5.7%) in the “repeat” and “single” tasks, respectively, following the intervention is similar to a previous report of a 10–13 cm improvement (no specific values are detailed) in developpé performance noticed following imagery use (Hanrahan and Salmela, 1990).

Although the mechanisms of effect of imagery, and DNI^TM^ in particular, are not fully revealed to date (Callow et al., 2013), DNI^TM^ may be associated with not only practicing existing motor plans and habits (Willems et al., 2009) but actually refining and ameliorating them, thus resulting in enhanced motor execution, as was noticed following the intervention. Another potential explanation for the noticed effectiveness of the DNI^TM^ intervention may lie in its emphasis on kinesthetic imagery, which was suggested by previous literature to benefit motor performance (Lotze, 2013) and tasks that emphsize the relationship between various segments of the body (e.g., pelvis vs. thigh) (Shenton et al., 2004; Giron et al., 2012). Furthermore, the empahsis of the DNI^TM^ intervention on anatomical-proprioceptive awareness of the hip joint could specifically benefit developpé performance, given its suggested role in controlling pelvic alignment (Gamboian et al., 2000; Kiefer et al., 2013).

The finding of significant increases in ankle height for the “repeat” and “single”, but not the “static”, tasks is similar to a findings of a previous study showing that ankle ROM during elevé performance improved for the “repeat”, and not “static”, task following motor imagery training in adolescent female dance students (Abraham et al., 2017). However, the reason for this is a subject for future studies.

The significant increases in hip flexion and abduction ROM in all three tasks following the intervention may suggest, as part of a motor learning effect, an improved use of the hip joint through imagery, potentially resulting in more proper, effective motor plan (Debarnot et al., 2014) and function and increased embodiment of hip anatomy and biomechanics, all leading to better motor control over the pelvic-hip complex, thus increasing ROM.

The amount of pelvic posterior tilt (range: 19.93°–23.89°) in the current study at both “pre” measurements in all three tasks is similar to the 15.8° and 22.8° reported in 5 novice and 5 skilled dancers, respectively (Wilson et al., 2004), and to the 16.2° in 8 skilled ballet dancers (Wilson et al., 2007), all while performing grand rond de jambe en l’air. It is also greater than the ∼5°–15° reported during performance of the plié movement (Gontijo et al., 2015). This may suggest that our participants exhibited sufficient pelvic posterior tilt at pre-intervention and therefore didn’t need to increase it. However, the lack of significant changes in pelvic posterior tilt ROM at “post”, suggests that participants were able to maintain pelvic alignment and control while increasing their hip ROM and gesturing leg height. Such motor strategy implies on a greater “hip-to-pelvis” ratio (Coker et al., 2015), suggesting a proper “pelvi-femoral rhythm” (Bohannon et al., 1985; Murray et al., 2002) and is advantageous for dance students by contributing to successful performance and enhancing technical and esthetic skill levels (Wilson et al., 2004, 2007) while serving as a protective mechanism against improper pelvic use and thus injuries (Bohannon et al., 1985; Murray et al., 2002; Deckert et al., 2007; Hagins, 2011; Franklin, 2014; Gildea et al., 2015; Gontijo et al., 2015). Our findings suggest that increasing leg height does not necessarily require increased pelvic motion. Thus, it may be that previous findings of increased pelvic ROM exhibited by skilled dancers in comparison to novices and of a positive correlation between pelvic ROM and leg height (Wilson et al., 2004, 2007) may be explained by the skilled dancers’ in these studies exhibiting difficulties in controlling and optimizing hip joint motion while trying to achieve higher level of performance, and potentially not using an ideal motor control strategy.

The amount of pelvic lateral tilt (i.e., hiking; range: 20.06°–25.42°) in the current study at both “pre” measurements in all three tasks is somewhat similar to the 20.9° and 30.7° of pelvic left tilt reported in 5 novice and 5 skilled dancers, respectively (Wilson et al., 2004), and to the 25.7°–38.1° reported for 8 skilled ballet dancers (Wilson et al., 2007), all while performing grand rond de jambe en l’air. This suggests that the participants exhibited a sufficient amount of pelvic lateral tilt at pre- intervention. Further, the lack of significant increase in pelvic hiking while increasing gesturing leg height is noteworthy, given pelvic hiking being commonly used by dancers as a compensatory strategy for achieving greater height of the gesturing leg or for compensating for lack of sufficient hip ROM (Watelain et al., 2001).

The significant decrease in knee extension in the “single” task suggest that participants followed the DNI^TM^ theme of using correct body biomechanics and succeeded in enhancing their developpé performance (i.e., increasing ankle height) without hyperextending the knee joint, a strategy often used by dance students for increasing the gesturing leg height although considered as a mechanisms of dance knee injuries (Quirk, 1983; Reid, 1988).

The significant improvements following the intervention in self-reported level of difficulty and proficiency in the “repeat” task and in self-reported amount of improvement in all three tasks (Table 6) along with the noticed kinematic improvements require further investigation before one can point at a correlation existing between objective and subjective (i.e., kinematic parameteres) measures of dance performance.

The findings of this study must be interpreted cautiously given several limitations. There was no long-term follow-up to investigate retention. Future studies should try to collect such data. Second, no assessments of participants’ engagement with the intervention and of participants’ hypermobility were conducted. Future studies should collect such data, potentially through questionnaires and hypermobility tests. Lastly, the lack of a control group and the participants serving as their own controls is another limitation due to the participants potentially being biased and trying harder to please the researchers. However, the fact that the DNI^TM^ trainers were not present during the measurement sessions likely minimized this potential source of bias.

In summary, this study adds to the evidence for the beneficial effect of DNI^TM^ for dance students and supports the notion that DNI^TM^ training is beneficial in improving dance performance and should be considered as an adjunct training method in dance training settings, with the goal of enhancing dance performance while maintaining the dancers’ physical and mental well-being and preventing injuries (Hanrahan and Salmela, 1990; Hanrahan, 1994, 1995; Hanrahan et al., 1995; Hanrahan and Vergeer, 2000; Heiland et al., 2012; Heiland and Rovetti, 2013; Pavlik and Nordin-Bates, 2016; Abraham et al., 2016, 2017). Unlike previous reports (Hanrahan and Salmela, 1990), the kinematic improvements in the current study were accompanied by improvements in participants’ self-reported observations. Future studies should explore the effect of DNI^TM^ on dancers’ cognitive and physical task demands and performance-related motor plans, including neural changes in the brain and the peripheral nervous system (Debarnot et al., 2014).

## Conclusion

The current study suggests that an intensive, 3 day DNI^TM^ training was effective in improving developpé performance by increasing hip ROM while maintaining correct pelvic alignment in University-level dance students. The intervention also resulted in gains in imagery ability and self-reported measures.

It provides additional evidence for the beneficial effect of a deliberate and designated application of imagery training on motor and non-motor aspects of dance performance. Further research is warranted for investigating the structured application of DNI^TM^ in dance training and performance and its associated mechanisms of effect.

## Author Contributions

AA and RG contributed conception and design of the study and collected the data. AA and BN organized the database and entered data. AA performed the statistical analysis and wrote the first draft of the manuscript. RS processed raw data. RG and MH contributed to manuscript revision. All authors read and approved the submitted version.

## Conflict of Interest Statement

AA is working in collaboration with the International Institute of the Franklin Method on developing DNI-related contents. The remaining authors declare that the research was conducted in the absence of any commercial or financial relationships that could be construed as a potential conflict of interest.
